# The therapeutic potential of different mesenchymal stem cells and their derived exosomes in metabolic dysfunction-associated steatotic liver disease

**DOI:** 10.3389/fendo.2025.1558194

**Published:** 2025-04-03

**Authors:** Dan Qin, Pingping Huang, Jialing Chen, Changjun Wu, Yuzhen Liang

**Affiliations:** ^1^ Department of Endocrinology, The Second Affiliated Hospital of Guangxi Medical University, Nanning, China; ^2^ Department of Respiratory and Critical Care Medicine, The Second Affiliated Hospital of Guangxi Medical University, Nanning, China

**Keywords:** metabolic dysfunction-associated steatotic liver disease, metabolic dysfunction-associated steatohepatitis, mesenchymal stem cells, exosomes, therapy

## Abstract

Metabolic dysfunction-associated steatotic liver disease is a metabolic disease with an increasing incidence. Its pathogenesis involves the interaction of multiple factors. There is currently no specific treatment, so early prevention and treatment are crucial. Mesenchymal stem cells are a type of cell with the ability to self-renew and differentiate in multiple directions. They have a wide range of sources, including umbilical cords, bone marrow, and fat, and have various biological functions such as anti-inflammation, immune regulation, anti-oxidation, and inhibition of fibrosis. They have shown significant potential in the treatment of non-alcoholic fatty liver disease. In recent years, mesenchymal stem cells derived exosomes have been shown to be rich in bioactive substances, and to be involved in intercellular communication, regulating metabolism, reducing inflammatory responses, improving lipid metabolism, inhibiting fibrosis, and other processes that contribute to the treatment of metabolic dysfunction-associated steatotic liver disease. Mesenchymal stem cells and mesenchymal stem cell-derived exosomes play an important role in the pathogenesis and treatment of metabolic dysfunction-associated steatotic liver disease and provide new potential and direction for the treatment of Metabolic dysfunction-associated steatotic liver disease. This article reviews the role and effects of mesenchymal stem cells and mesenchymal stem cell-derived exosomes from different sources in Metabolic dysfunction-associated steatotic liver disease and discusses their prospects as potential therapeutic strategies.

## 1 Introduction

Metabolic dysfunction-associated steatotic liver disease (MASLD) is the most common chronic disease with a prevalence approaching 25% worldwide (1–3). The disease was once known as non-alcoholic fatty liver disease (NAFLD), in 2023, a consensus panel led by the Liver Association proposed to redefine NAFLD as MASLD (4). This new definition better reflects the pathophysiological characteristics of fatty liver and metabolic abnormalities and provides a new perspective for clinical diagnosis and treatment. MASLD is a clinical and pathological syndrome with no history of excessive alcohol consumption, multiple causes, and characterized by lipid accumulation and fat degeneration in the liver (5, 6). Studies have shown that about 3% to 5% of patients with fatty liver disease can develop Metabolic dysfunction-associated steatohepatitis (MASH), which is characterized by liver inflammation and hepatocellular injury (6). As the disease progresses, hepatic steatosis can further develop into MASH and liver fibrosis, ultimately leading to cirrhosis and hepatocellular carcinoma (HCC) (7). As a complex metabolic disease, there is currently no specific treatment for MASLD (8). Lifestyle interventions are still recognized as the first choice of treatment. In addition, drug interventions can regulate glucose and lipid metabolism, mitigate inflammation, and delay liver fibrosis progression, providing an effective strategy to slow the development of MASLD (9).

Mesenchymal stem cells (MSCs) are a type of pluripotent progenitor cell with the ability to self-renew and differentiate into multiple lineages, including adipocytes, osteocytes, osteoblasts, and chondrocytes (10–12). Initially, MSCs were isolated from bone marrow mesenchymal stem cells (BM-MSCs) (BM-MSCs) (13). With the deepening of research, it was gradually discovered that MSCs also exist in adipose tissue,skeletal muscle, umbilical cord blood, lung and other tissues (14–17). MSCs from different sources have various biological functions such as anti-inflammatory, anti-oxidative, immune regulation and tissue regeneration, which makes them show unique advantages in the treatment of various diseases (18, 19). In recent years, mesenchymal stem cell-derived exosomes (MSCs-Exo) have become a research hotspot because it contains a variety of bioactive substances. Exosomes are small extracellular vesicles with diameters ranging from 30 to 150 nanometers, which are secreted by various types of cells, including stem cells, immune cells, adipocytes, hepatocytes, tumor cells, etc. (20–28). Exosomes are generated through the inward budding of the cell membrane, resulting in the formation of vesicles, which contain biologically active substances such as nucleic acids, proteins and growth factors. They play a crucial role in intercellular communication and signal transduction by merging with the cell membrane and releasing their contents into the extracellular space (29). They reach distant cells and tissues through body fluids such as blood, lymphatic fluid and saliva to regulate metabolic processes in the body (30–33). An increasing number of studies have shown that exosome-mediated drug delivery has the advantages of low toxicity, low immunogenicity and high engineering, and has broad application prospects in the treatment of diseases in the future. This article will summarize the role and therapeutic potential of microRNAs derived from MSC- Exo, especially those derived from mesenchymal stem cells, in MASLD.

## 2 The conventional pathogenesis of metabolic dysfunction-associated steatotic liver disease

The pathogenesis of MASLD involves the interaction of multiple factors, including insulin resistance (IR), excessive accumulation of fatty acids, oxidative stress, inflammatory response, intestinal microbiota imbalance, adipose tissue and mitochondrial dysfunction, and liver fibrosis (34, 35). With the innovation of detection technology and the emergence of new research results, the “second hit” hypothesis proposed by James and Day can no longer summarize the complex and ever-changing pathogenesis of MASLD (34). However, the emergence of new theories such as the “three-hit hypothesis” in recent years has well compensated for the deficiencies of the “two-hit” theory, which includes steatosis, lipotoxicity and inflammation (6, 36). IR is one of the core pathogenesis mechanisms of MASLD (37). Insulin normally inhibits lipolysis in adipose tissue. However, when the sensitivity of insulin-targeted organs (such as the liver, muscle and adipose tissue) to insulin decreases, the inhibitory effect of insulin is weakened, leading to fat accumulation in the liver. IR leads to increased fat accumulation in the liver by stimulating lipolysis and inducing hyperinsulinemia, thereby setting the stage for the excessive buildup of fatty acids (38).

Excessive accumulation of fatty acids in the liver is a key link in the progression of MASLD. In the context of insulin resistance, the breakdown and oxidation of fatty acids increases, leading to many free fatty acids entering hepatocytes, promoting lipotoxicity and mitochondrial dysfunction in hepatocytes, thereby triggering hepatocyte apoptosis. These fatty acids cannot be completely oxidized, thus accumulating in the liver and forming steatosis (39, 40). Excessive accumulation of fatty acids not only increases the pressure inside liver cells, but also provides conditions for the occurrence of oxidative stress and inflammation. In addition, dysfunction of adipose tissue can lead to an imbalance of adipokines, such as abnormal secretion of leptin and adiponectin, which exacerbates lipid accumulation in the liver. At the same time, these adipokines play a critical role in mediating the inflammatory response and driving the fibrosis process (41). Excessive accumulation of fatty acids and abnormal metabolism directly lead to increase in reactive oxygen species (ROS) in liver cells, which activates oxidative stress (42). Oxidative stress exacerbates the inflammatory response in the liver by damaging liver cells, promoting the peroxidation of fatty acids, and increasing the release of cytokines, leading to chronic low-grade chronic inflammation, infiltration of immune cells such as adipocytes, macrophages, and T cells, and production of inflammatory mediators such as cytokines and chemokines. These inflammatory reactions further promote cell damage and fibrosis in the liver, which may eventually progress to cirrhosis or hepatocellular carcinoma (43, 44).

The final stage of MASLD is liver fibrosis, which is usually caused by an imbalance between hepatocyte death and reduced regenerative capacity. Persistent harmful factors (such as oxidative stress, inflammation, and intestinal microbiota imbalance) cause activation of hepatic stellate cells, which in turn leads to excessive extracellular matrix accumulation, ultimately leading to changes in liver structure, accompanied by fibrosis, regenerative nodules, and inflammatory cell infiltration. Although the current treatment of choice for MASLD is lifestyle intervention (e.g., diet, exercise, and weight loss), there is still a lack of specific drugs for MASLD (9). New therapeutic strategies focus on mitigating pathological processes, including lipid accumulation, inflammation, and oxidative stress in the liver. In addition, novel therapies based on non-coding RNAs, and MSCs-Exo are becoming a research hotspot and hold the potential to bring new breakthroughs in the treatment of MASLD.

## 3 MSCs in MASLD

**Figure 1:**
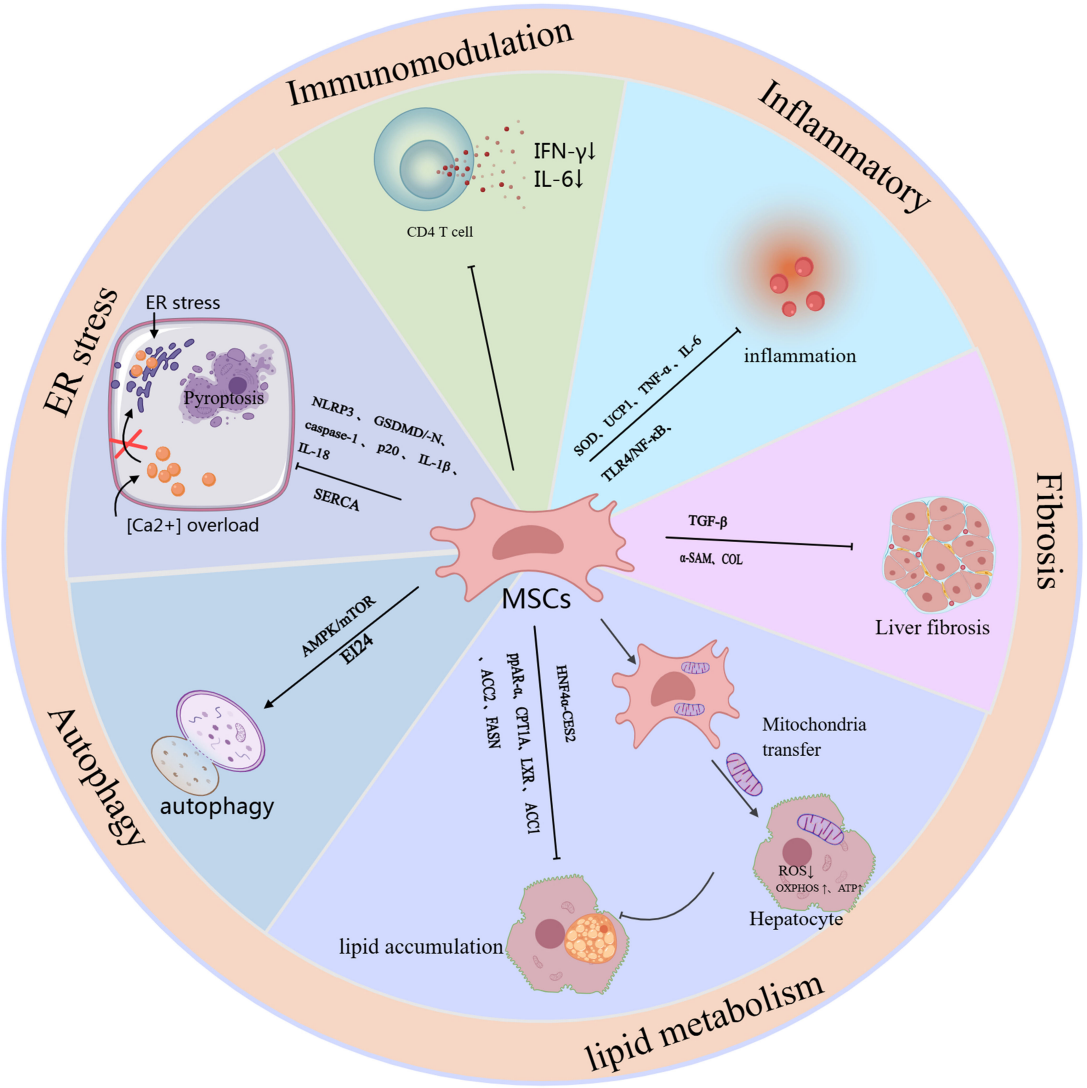
The schematic role of MSC in MASLD. Mechanisms and pathways of mesenchymal stem cells (MSCs) in the treatment of MASLD. This figure outlines the mechanisms and signaling pathways by which MSCs exert their therapeutic effects, specifically targeting liver function to alleviate MASLD, and it outlines the mechanisms and pathways associated with the therapeutic efficacy of different sources of MSCs and combination therapies.

Mesenchymal stem cells (MSCs) affect the occurrence and development of MASLD through multiple pathways. The main mechanisms include: (1) improving metabolic disorders; (2) reducing inflammation and oxidative stress; (3) antifibrotic effects; (4) inducing autophagy; and (5) immunosuppressive effects (**Figure 1**).

### 3.1 Adipose-derived mesenchymal stem cells in MASLD

ADSCs show great potential in the research and treatment of MASLD due to their rich sources, easy availability, and low impact on the body. Studies have shown that ADSCs can effectively improve the symptoms of MASLD through mechanisms such as regulating lipid metabolism, reducing inflammation, relieving oxidative stress, and anti-fibrosis. For example, Watanabe et al. found in a Mc4r-KO knockout MASH mouse and a LPS-induced MASH mouse model that ADSC intervention significantly reduced the levels of serum ALT and inflammatory markers, while increasing the proportion of anti-inflammatory macrophages in the liver, providing a new basis for the treatment of MASLD (45). Saleh et al. also studied obesity-related MASLD and showed that ADSC treatment can reverse liver fat accumulation in obese mice and decrease TNF-α and IL-6 expression levels, improving MASLD symptoms (46). In addition to reducing inflammation, antifibrosis is also an important target in the treatment of MASLD (47). Yano et al. found that in a mouse model of MASH, u-ADSCs were able to reduce liver inflammation and cell infiltration and showed similar therapeutic effects to wild-type u-ADSCs in terms of fibrosis. In particular, MASH (12 weeks) u-ADSCs performed better in regulating metabolism and intracellular transport, while MASH (4 weeks) u-ADSCs were more significant in reducing inflammation (48). In addition, the therapeutic potential of modified ADSCs in MASLD has also received widespread attention. For example, Domingues et al. used an adenovirus to construct the antioxidant gene Sod2 and upregulate it in ADSCs. The study found that modified ADSCs significantly improved the liver fat content of obese mice, mainly by reducing liver inflammation and oxidative stress (49). This was also verified in an *in vivo* experiment by Afarin et al., who found that by injecting LPS-stimulated ADSCs into injecting LPS-stimulated ADSCs into a MASLD rat model, it was found that they not only effectively corrected liver enzyme levels (such as ALT and AST), it not only significantly decreased the expression of transforming growth factor β (TGF-β) and inflammation-related genes but also lowered the levels of ROS, demonstrating the potential of ADSCs in the treatment of MASLD (50).

### 3.2 Role of umbilical cord mesenchymal stem cells in MASLD

UCMSCs, as another source of mesenchymal stem cells, are not only easy to obtain, but also exhibit stronger differentiation potential and lower immune rejection compared to mesenchymal stem cells from other sources, making it a potential therapeutic strategy for treating MASLD. In particular, human-derived umbilical cord mesenchymal stem cells (HUCMSCs) have attracted widespread attention for their use in MASLD. Existing studies have shown that hUCMSCs mainly function in the treatment of MASLD through mechanisms such as anti-inflammation, anti-fibrosis, autophagy induction, regulation of lipid metabolism, and improvement of insulin resistance. In a mouse model of MASLD, HUCMSCs significantly improved liver function and reduced lipid deposition by tail vein injection, and improved lipid metabolism by upregulating the HNF4α-CES2 pathway and regulating genes related to fatty acid oxidation (51). Impaired autophagy is another important mechanism of MASLD (52). Studies have found that HUCMSCs can promote autophagy by suppressing the expression of mTOR and P62, and promoting the levels of AMPK and LC3BII/α, thereby accelerating the degradation of body fat and alleviating the symptoms of MASLD (53). In addition, HUCMSCs also improve fat metabolism and reduce inflammatory responses by regulating the PPAR signaling pathway, enhancing the expression of PPAR-α, and targeting CPT1A. Further studies found that in mice treated with HUCMSCs, the expression of α-SMA protein and various fibrosis-related collagens (such as Col1a1, Col1a2, and Col3a1) was significantly downregulated, reducing the degree of liver fibrosis (54). In recent years, researchers have explored the potential of combining HUCMSCs with other treatments to further improve the therapeutic effect of MASLD. For example, one study showed that the combination of HUCMSCs and liraglutide significantly improved liver function damage and pathological changes in rats with MASLD associated with type 2 diabetes mellitus (T2DM). After 8 weeks of combined treatment, HbA1c, HOMA-IR, ALT and AST levels were significantly reduced, and glucometabolic, insulin resistance and liver damage were significantly improved. At the same time, they inhibited the TLR4/NF-κB inflammatory pathway, resulting in a significant reduction in the expression of IL-6, TNF-α, and the antioxidant enzyme superoxide dismutase (SOD). These results suggest that combination therapy with hUCMSCs and other drugs may be an effective strategy to enhance the therapeutic effect, providing new ideas for the clinical management of MASLD (55).

### 3.3 Role of bone marrow mesenchymal stem cells in MASLD

BMSCs are one of the most extensively researched types of MSCs. Similar to other MSC types, BMSCs have the advantages of being easy to obtain, having multi-directional differentiation potential and low immunogenicity. They are also ideal for treating a variety of diseases because they do not involve ethical issues and can be autologous transplanted. Existing studies have shown that BMSCs can significantly improve MASLD through multiple mechanisms, such as anti-inflammation, anti-fibrosis, inhibition of T cell proliferation, alleviation of mitochondrial dysfunction, alleviation of endoplasmic reticulum stress, and regulation of glycolipid metabolism.The study shows that that BMSCs have a significant role on obese mice. Compared with untreated mice, BMSCs-treated mice can effectively prevent the occurrence of liver fibrosis. This is manifested in the fact that the gene expression levels of inflammatory factors and fibrosis markers in the liver (such as IL-1β, INF-γ, TNF-α, TGF-β1 and collagen type I) are close to normal or even lower than normal, thereby reducing liver tissue damage (56).

Endoplasmic reticulum (ER) stress is considered an important trigger of MASLD (57). Li et al. found that BMSCs intervention can improve steatosis, insulin resistance and dyslipidemia, and further revealed its potential molecular mechanism. Specifically, BMSCs alleviate endoplasmic reticulum stress in MASLD rats and PA-induced HepG2 cells by regulating SERCA (calcium pump) to restore intracellular Ca²^+^ homeostasis, inhibit cell pyroptosis, and improve metabolic dysfunction (58). In recent years, MSC has gradually become an emerging way to improve disease progression through the mitochondrial transfer mechanism. Bi et al. found that BMSCs significantly reduced ROS production and enhanced oxidative phosphorylation (OXPHOS) activity and ATP production by transferring mitochondria to fat cells, thereby effectively maintaining the cell’s energy balance and restoring liver function. This mechanism can inhibit the inflammatory response and lipid and glucose metabolism disorders associated with MASLD (59). Nickel et al. further verified this finding in their study, which showed that by transplanting the mitochondria of human BMSCs into the hepatocytes of MASH mice, the lipid decomposition capacity can be enhanced, thereby significantly improving the lipid load of MASH mice (60). This innovative mechanism repairs damaged cells through mitochondrial transfer, independent of cell differentiation or paracrine effects, providing new research direction for cell therapy.

### 3.4 Role of mesenchymal stem cells from other sources in MASLD

In addition to the aforementioned common adipose tissue-, bone marrow- and umbilical cord-derived MSCs, other sources of MSCs, such as bone-derived MSCs and menstrual blood-derived endometrial stem cells (MenSCs), have also shown potential in the treatment of MASLD. For example, Wang et al. showed that bone-derived MSCs inhibit the proliferation of CD4+ T cells through immunomodulatory effects, thereby reducing inflammation in MASLD (61). In addition, they conducted another study to demonstrate that in a mouse model of MCD diet-induced MASH, this protective effect may be related to the activation of bone-derived MSCs to inhibit the secretion of IFN-γ and IL-6 by CD4+ T cells (62). MenSCs, on the other hand, target the AMPK-mTOR signaling pathway by secreting the novel regulatory factor Rnf186, which improves lipid metabolism and insulin resistance in MASLD mice, thereby alleviating the symptoms of MASLD (63). In summary, mesenchymal stem cells of different origins affect the development and progression of MASLD through multiple mechanisms, providing more options for the future treatment of MASLD. Future research will further explore the therapeutic potential of different MSC sources and promote their clinical application.

In summary, mesenchymal stem cells from different sources have their own advantages in the treatment of MASLD and can influence the onset and progression of MASLD through distinct mechanisms. This provides more treatment choices for MASLD, especially in the context of individualized treatment. The appropriate source of MSCs can be selected according to specific circumstances to achieve more precise and effective treatment. At present, there are still many gaps in the application of MSCs from other sources in the treatment of MASLD, and their specific mechanisms need to be further explored to promote the clinical application of MSCs in treating MASLD (**Supplementary Table 1**).

## 4 The role of MSCs-Exo in MASLD

**Figure 2:**
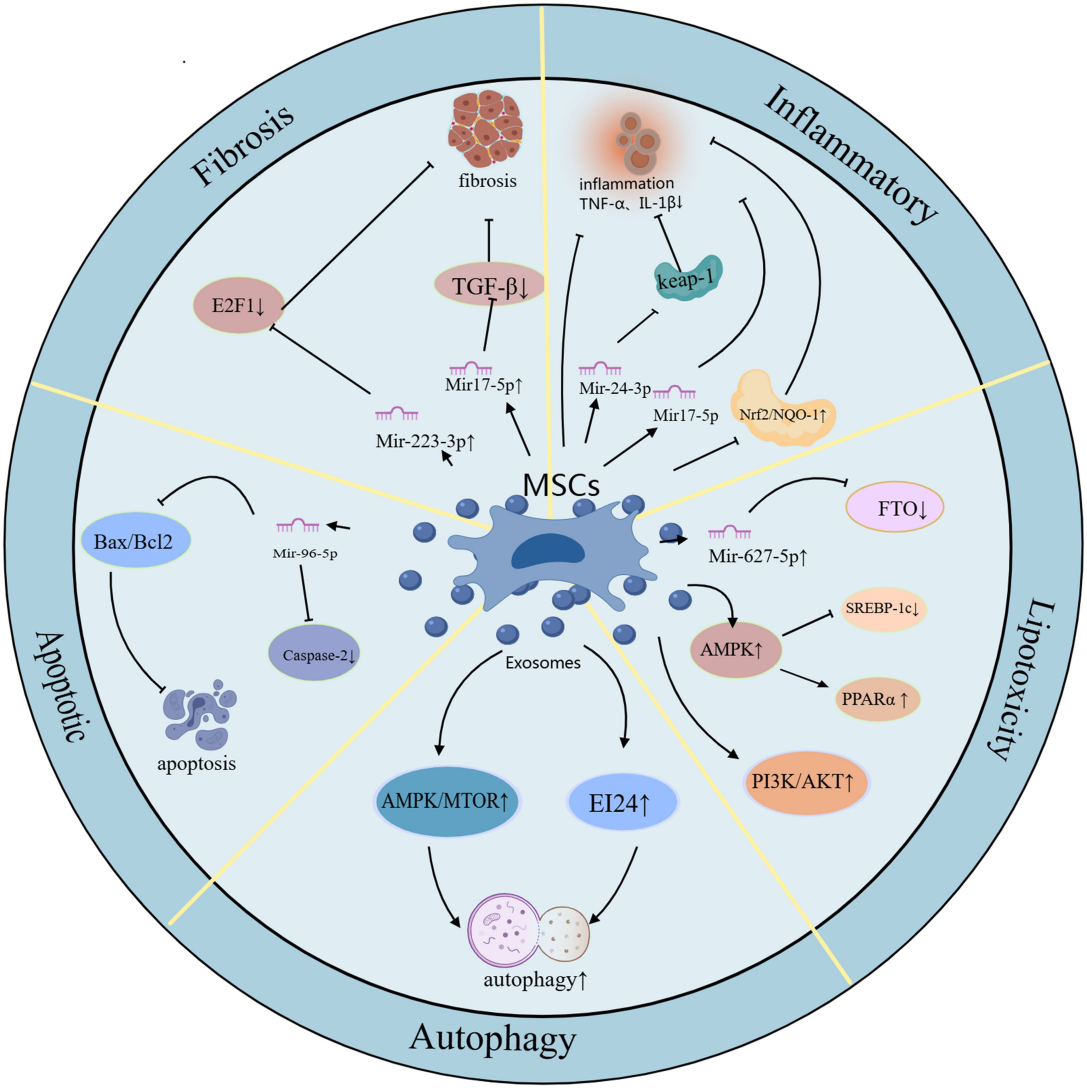
The schematic role of MSC exosomes in MASLD. Mechanisms and pathways of exosomes derived from different MSCs in the treatment of MASLD therapy. Exosomes derived from different MSCs have anti-inflammatory, oxidative stress inhibiting, lipid metabolism regulating, apoptosis inhibiting, autophagy inducing, and liver fibrosis attenuating effects to protect MASLD.

MSCs-Exo play an important role in the treatment of MASLD, mainly through the following mechanisms: (1) inhibiting oxidative stress and inflammatory response; (2) regulating glycolipid metabolism; (3) inhibiting apoptosis and inducing autophagy; (4) alleviating liver fibrosis (**Figure 2**).

### 4.1 Adipose-derived mesenchymal stem cell-derived exosomes and MASLD

In recent years, ADSC-Ex has emerged as a novel therapeutic strategy that has unique advantages in the occurrence, development and treatment of MASLD. Number of studies have shown that ADSC-Exo not only affects the onset and progression of MASLD through multiple pathways, but also effectively relieves the symptoms of MASLD, including regulating glycolipid metabolism, inhibiting inflammatory response and liver fibrosis. Abnormal liver lipid metabolism and abnormal accumulation of lipids are key factors in the development of MASLD. Studies have found that ADSC-Exo can regulate lipid metabolism in multiple ways to reduce lipid accumulation and thereby improve MASLD symptoms. For example, a study by Qinghui Niu et al. showed that ADSC-Exo carrying miR-223-3p can target and inhibit the expression of E2F1, thereby inhibiting lipid accumulation and liver fibrosis and improving MASLD symptoms (64). In addition, studies have indicated that adipose tissue plays a key role in a number of diseases associated with insulin resistance, particularly MASLD. Togliatto et al. found that ADSC-Exo derived from obese individuals may have therapeutic potential in the process of angiogenesis, and also showed that ADSC-Exo plays an important role in obesity-related metabolic complications (65). A Baranova et al. believe that miRNAs such as miR-122 and other miRNAs may play a positive role in preventing MASLD -related hepatocellular carcinoma, and MSC-derived exosomes derived from adipose tissue are an ideal vehicle for these miRNAs (66). Qinghui Niu’s research also showed that miR-223-3p carried by ADSC-Exo can delay the progression of MASLD, suggesting that ADSC-Exo loaded with miR-223-3p may be a potential strategy for the treatment of MASLD.

### 4.2 Human umbilical cord mesenchymal stem cell-derived exosomes and MASLD

HUCMSCs-Exo is an exosome with a wide range of sources and low immunogenicity. Meanwhile, current research shows that PPARα can improve steatosis, inflammation and fibrosis, and is a potential new therapeutic target for MASLD (67). Studies have shown that hUCMSCs-Exo can significantly improve the symptoms of MASLD by regulating lipid metabolism, reducing inflammation, and reducing oxidative stress, inhibiting apoptosis, inducing autophagy, and inhibiting liver fibrosis. These functions mainly depend on the miRNA carried by it to regulate related signal pathways. In the early stages of MASLD, abnormal lipid deposition and steatosis are its main pathological features (68). HUCMSCs-Exo improves MASLD by regulating lipid metabolism to reduce lipid accumulation. Fuji Yang et al. showed that h UCMSCs-Exo can significantly reduce lipid accumulation and improve hepatic steatosis by activating the AMPK-dependent pathway to inhibit SREBP-1c-mediated fatty acid synthesis and enhance peroxisome proliferator-activated receptor (PPARα) mediated fatty acid oxidation (69). In addition, Lidan Cheng and others found that miR-627-5p carried by hUCMSCs-Exo can inhibit the expression of genes associated with fat synthesis,(such as G6Pc, PEPCK, FAS and SREBP-1c) in palmitic acid-treated L-O2 cells, while upregulating the expression of PPARα, thereby improving glycolipid metabolism and reducing lipid accumulation (70). Inflammation and oxidative stress are key mechanisms in the development of MASLD, and hUCMSCs-Exo also showed significant efficacy in inhibiting inflammation and oxidative stress. Studies have shown that hUCMSCs-Exo alleviates inflammatory and oxidative stress responses by reducing the secretion of TNF-α and IL-6,and activating the Nrf2/NQO-1 antioxidant signaling pathway (71). In addition, miR-24-3p in hUCMSCs-Exo can further alleviate inflammation and oxidative stress by targeting and inhibiting Keap-1 to reduce ROS production (72).

As MASLD progresses, liver fibrosis becomes another key pathological feature. Recent studies have found that hUCMSCs-Exo can effectively inhibit liver fibrosis and delay the progression of MASLD through the anti-fibrosis miRNAs it carries. For example, Sani et al. showed that anti-miR-17-5p-enriched hUCMSCs-Exo can inhibit the progression of liver fibrosis by downregulating the expression of TGF-β1, IL-1β, and IL-6, thereby reducing the accumulation of extracellular matrix (73). Exosomes can enhance hepatocyte autophagy through the AMPK/mTOR or ei24-related autophagy pathway, thereby reducing liver fat and collagen deposition (53). In addition, He et al. found that hUCMSCs-Exo can promote the formation of autophagosomes in T2DM rats and palmitic acid-treated L-O2 cells, and improve glycolipid metabolism through the autophagy pathway (74). It is worth noting that Tawfeek et al. found that curcumin-pretreated hUCMSCs-Exo improved lipid accumulation and reduced liver inflammation and oxidative stress in MASH mice. This study shows that the pretreated hUCMSCs-Exo further enhances its efficacy, providing greater potential for clinical treatment (75). These studies provide an important theoretical basis and new research directions for the future clinical treatment of MASLD, and provide more possibilities for the clinical application of MSCs-Exo.

### 4.3 Bone marrow mesenchymal stem cell-derived exosomes and MASLD

BM-MSCs are one of the most used sources of MSCs. Recent studies have shown that BM-MSCs-Exo also play an important role in the treatment of MASLD. BM-MSCs-Exo alleviate the progression of MASLD through multiple pathways, including anti-apoptosis, improving lipid metabolism, enhancing mitochondrial autophagy, anti-inflammation and improving insulin resistance. Insulin resistance is the “first blow” in the hypothesis of the pathogenesis of MASLD. Studies have found that BM-MSCs-Exo can alleviate the progression of MASLD by activating the PI3K/AKT signaling pathway, improving IR and lipid droplet accumulation, and promoting the expression of glucose transporter 4 (GLUT4) (76). In addition, BM-MSCs-Exo can improve steatosis in MASH rats by regulating lipid metabolic disorders. For example, El-Derany et al. found that BM-MSCs-Exo treatment significantly downregulated the expression of genes related to fatty acid synthesis and uptake, significantly upregulated the expression of genes related to fatty acid oxidation, and improved the steatosis of MASH (77). BM-MSCs-Exo can also reduce the Bax/Bcl2 ratio in the liver of MASH rats and protect hepatocytes from apoptosis through its anti-apoptotic effect. Further studies have shown that BM-MSCs-Exo can alleviate the effects of a high-fat diet on the liver by activating the expression of mitochondrial autophagy genes (77). In addition, miRNA-96-5p plays an important role in BM-MSCs-Exo, and its regulatory mechanism is closely related to the therapeutic effect of MASLD.

### 4.4 Other mesenchymal stem cell-derived exosomes and MASLD

In addition to the above sources, other types of MSC-Exos (such as MSC-Exo derived from umbilical cord blood, placenta, and dental pulp) also show good prospects in the treatment of MASLD. Although these studies are still in their infancy, there is growing interest in their potential therapeutic effects on MASLD. This is confirmed by the study by Chang, Chao-Yuan et al., which demonstrated that treatment with exosomes derived from human placental chorionic mesenchymal stem cells can reduce inflammation and inhibit the expression of NF-κB and HIF-1α in the liver tissue of obese mice (78). In addition, let-7i-5p miRNA is highly expressed in exosomes derived from human placental chorionic mesenchymal stem cells. Studies of its associated mechanisms have found that it mediates the treatment of obesity-associated sepsis, but whether it plays a role in the treatment of obesity-associated MASLD is unknown (**Supplementary Table 2**).

## 5 Clinical evidence and challenges

**Figure 3:**
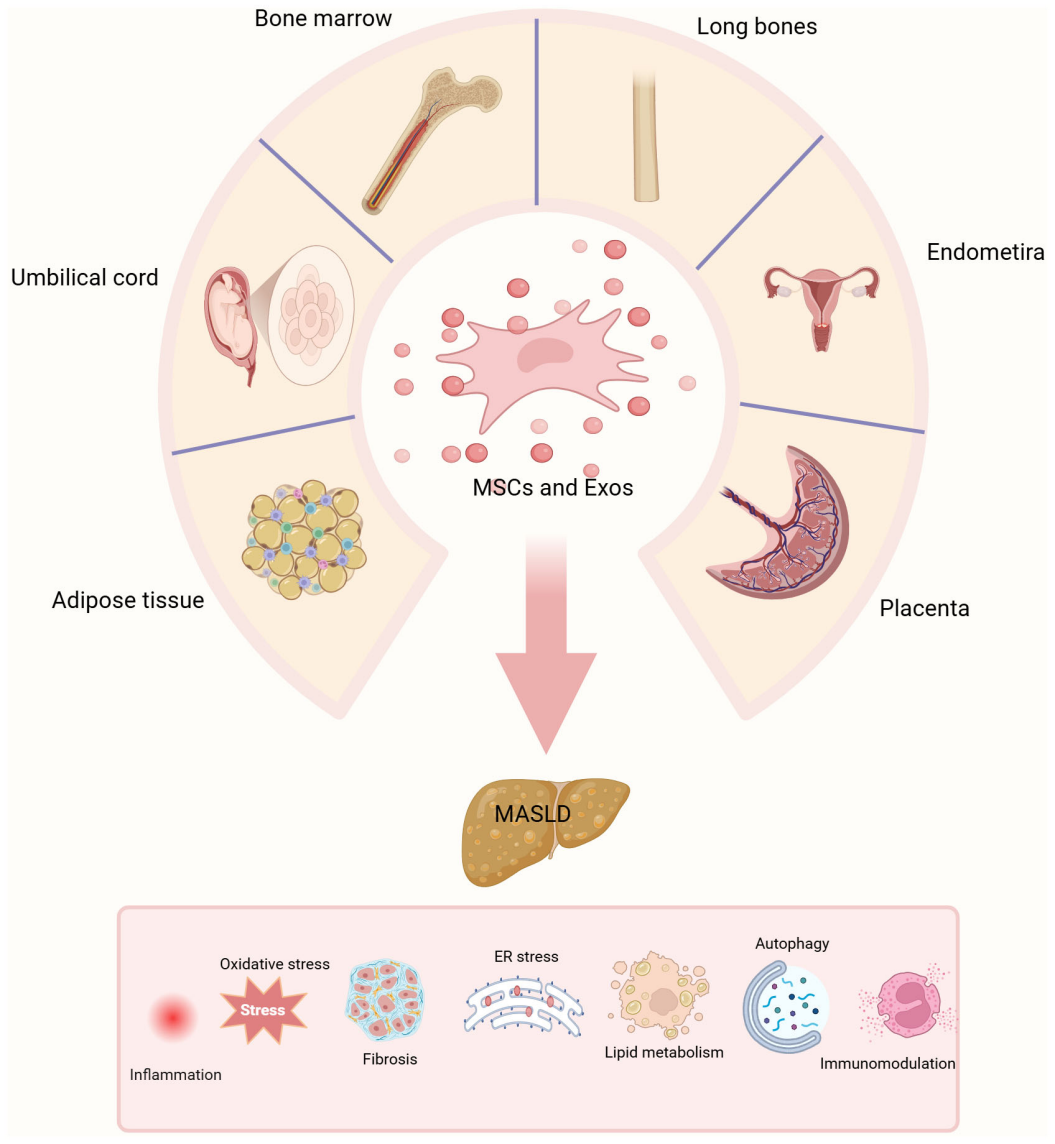
Graphical abstract. The different sources of MSCs and MSCs-exo. MSCs are harvested from various tissue sources, including adipose tissue, bone marrow, umbilical cord, endometrium, long bones, and placenta. This figure illustrates the evaluation of the therapeutic potential of MSCs and their exosomes.

MSC-Exo has important potential in the treatment of MASLD. The above studies have shown that MSC-Exo improves the symptoms of MASLD through mechanisms such as regulating glycolipid metabolism, inhibiting inflammation and oxidative stress, inducing autophagy, and reducing fibrosis (**Figure 3**). Compared with traditional MSC therapy, MSC-Exo, with its low immunogenicity, small size, and higher biosafety, provides a new idea for precision intervention in MASLD. However, current research on MSC-Exo for the treatment of MASLD is still in the experimental stage, and its clinical translation still faces many challenges. Studies have shown that there are differences in the efficacy of MSCs and MSC-Exos from different sources (fat, umbilical cord, bone marrow, etc.), which may be related to donor factors (age, health status), culture conditions, and exosome isolation methods (ultracentrifugation, size exclusion chromatography, etc.) (79, 80). In addition, there is a lack of uniform standards for the isolation and characterization of MSC-Exo, which affects the comparability between different studies and poses certain challenges in assessing the optimal applicability of different MSC sources (81). Therefore, establishing a standardized MSC isolation, culture and delivery protocol is crucial to improving its clinical translation. In addition, current studies on MSCs and MSC-Exo in the field of MASLD generally have small sample sizes, lack randomized controlled trials, and have few primate models and clinical investigations, which are mostly limited to animal models and *in vitro* experiments. Although existing animal models (such as obesity models) and *in vitro* models can simulate some of the disease characteristics of MASLD that are similar to those in humans, the effects of *in vivo* and *in vitro* studies cannot be directly and effectively applied to the clinic because animal models and *in vitro* models differ from humans in terms of metabolism, immunity and disease development. This step limits their clinical application to a certain extent.

The safety and efficacy of MSCs and MSC-exos in different MASLD patient populations still need to be determined by robust clinical trials (82). Clinical studies of MSC and MSC-Exo therapy for MASLD are still in their early stages. Most of these studies are still in phase I or II, and there are no large-scale phase III studies yet. Therefore, only short-term effects can be used as a reference, and the long-term efficacy and safety need to be verified (83). As pointed out by Jinag et al., the long-term safety of MSC and MSCs-Exo therapy is also a key issue (84). Although MSC and MSC-Exo did not cause significant rejection or tumorigenesis in early clinical studies, MSCs have a certain degree of plasticity. When MSCs and MSC-Exo are used for a long time or in high doses, we still need to fully evaluate the risk of their possible unintended differentiation (such as fibroblast-like differentiation), immune rejection, tumorigenesis, and long-term adverse effects on liver tissue (85, 86). A meta-analysis showed that hUCMSC therapy can improve glucose metabolism and insulin secretion in T2DM, providing some hope for hUCMSC therapy for DN (87). This was also confirmed by another meta-analysis study, which reported that MSC and MSCs-Exo therapy is a relatively safe treatment that can significantly improve liver function compared to conventional therapy (88). However, the inherent heterogeneity of MSCs results in significant variability in the therapeutic effects of these cells depending on their source (bone marrow, adipose tissue, or umbilical cord) and the methods used for isolation and preparation. Therefore, strategies to improve their safety, including but not limited to autologous MSC transplantation, immunosuppressive therapy, and gene editing techniques, are being explored to reduce their potential therapeutic risks. In addition, MSC and its MSC-Exo are biological agents, and various countries have strict regulatory requirements for the use of MSC-Exo in disease treatment, which is a major challenge to be solved in the future. Although MSC and MSCs-Exo have great potential in the treatment of MASLD, there is a lack of uniform standards for MSC and MSCs-Exo isolation protocols, effective doses, efficacy, cargo content, and information on their heterogeneous populations, making it difficult to draw conclusions (89). These issues pose major obstacles to current research on MSCs and their derived exosomes, and need to be addressed through further research. In the future, to promote the clinical application of MSCs and MSCs-Exo in the treatment of MASLD, in addition to the above challenges, optimizing miRNA delivery strategies is also an important issue. Enhancing the expression of specific miRNAs through genetic engineering or using engineered exosomes to improve the efficiency of targeted delivery can enhance their therapeutic effects (90). At the same time, exploring biomarkers that can predict efficacy is also a key direction, such as exosomes protein or miRNA indicators in the blood circulation, in order to screen for patients who are more suitable for MSC-Exo treatment and achieve personalized medicine (91). In addition, the production efficiency and quality of exosomes still face technical challenges. In the future, the yield and quality control can be improved by microfluidic chip separation or bioreactor culture technology to meet clinical needs (92).The above issues are the main challenges currently facing the research on MSCs and their derived exosomes. Future research needs to address these challenges and conduct large-scale, multi-center clinical studies. Integrating MSC and MSCs-Exo therapies into clinical practice can provide new hope for the effective treatment of MASLD patients and improve patient outcomes.

## 6 Conclusion

As mentioned above, the progression of MASLD involves multiple pathogenesis mechanisms, including the result of the combined effects of various factors such as environment and genetics. We summarize the research progress of the effects of MSCs and MSCs-Exo on MASLD and provide a brief overview of the involved mechanisms. MSCs and MSCs-Exo are important for the treatment of MASLD due to their differentiation ability and other promising properties, by inducing anti-inflammation, improving lipid metabolism, enhancing insulin sensitivity, balancing oxidation and anti-fibrosis. However, there are still many gaps that need to be further explored and filled. In addition, the preparation, quality control and safety of MSCs and MSCs-Exo are all practical and important issues that will face future clinical applications. In the future, promoting the application of MSCs and MSCs-Exo in the treatment of MASLD will require optimizing miRNA delivery, screening for biomarkers to achieve personalized treatment, and improving production efficiency and quality control of exosomes to meet clinical needs. In summary, MSCs and MSCs-Exo have great potential in the treatment of MASLD. We need to continue to explore and improve the therapeutic strategies of MSCs and their derived exosomes for the treatment of MASLD. This will provide more effective, safe and reliable options for the prevention and treatment of MASLD.
