# Supplementary material for: The therapeutic potential of different mesenchymal stem cells and their derived exosomes in metabolic dysfunction-associated steatotic liver disease
Source: Front Endocrinol (Lausanne). 2025 Apr 3;16:1558194. doi: 10.3389/fendo.2025.1558194 (PMC12003127; doi:10.3389/fendo.2025.1558194)
Supplement: Supplementary file 1 [file Table1.docx]

Supplementary Material

**Supplementary Table 1.**

| **Table 1. Effects of MSCs in MASH/MASLD** | | | | | | | | |  |
| --- | --- | --- | --- | --- | --- | --- | --- | --- | --- |
| **Name** | **Animal model** | **In vitro model** | **Precondition** | **Combination** | **Route of administration** | **Dose/cell** | **Follow-up period** | **Results** | **References** |
|  |  |  |  |  |  |  |  |  |  |
| hUC-MSC | C57BL/6 mice |  |  |  | intravenous injections | 1.5 × 106 cells | 8weeks | Improved insulin sensitivity  Alleviated steatosis and inflammation  Decreased expression of α-SMA protein and fibrosis-related collagen (COL) genes (Col1a1, Col1a2 and Col3a1), alleviated liver fibrosis | (54) |
| HucMSCs |  | AML12cells |  |  | co-culture | 2 × 106 cells | 24-48h | Promotes lipid degradation and reduces lipid synthesis  Regulates the AMPK/MTOR pathway and activates autophagy  EI24 is significantly overexpressed | (53) |
| hUC-MSCs | Sprague Dawley rats |  |  | Liraglutide (200 μg/kg) | intravenous injection | 1 × 106 cells | 8weeks | Reduce inflammation and oxidative stress  Improve glucose and lipid metabolism and insulin resistance  Down-regulate the TLR4/NF-κB inflammatory pathway | (55) |
| hUC-MSCs | C57BL/6 db/db mice |  |  |  | intravenous injections | 1 × 106 cells | 6weeks | Up-regulation of genes involved in fatty acid β-oxidation (PPARα and its target genes ACOX1, Angplt4 and Cpt1b)  Down-regulation of genes involved in lipogenesis (LXR, ACC1, ACC2 and FASN)  Up-regulation of the HNF4α-CES2 pathway | (52) |
| ADSCs | C57BL/6J mice |  | Overexpression of SOD2 |  | intraperitoneal injection | 1.5× 106 cells | 4weeks | Upregulates Sod2 and UCP1 expression  Reduces inflammation and oxidative stress  Improves glucose tolerance and fat accumulation | (49) |
| AD-MSC | C57BL/6J *Mc4r-KO* mice |  |  |  | intravenous injections | 1 × 106 cells | 4weeks | The number of anti-inflammatory macrophages (CD11b + F4/80 + Ly6c low) increases  and reduces inflammation (IL6, TNF-α) and fibrosis. | (45) |
| ASCs | C57BL/6 mice |  |  |  | intraperitoneal injection | 4.2 × 107 cells | 21weeks | Inhibits fat degeneration and lipid accumulation  Downregulates TNF-α and IL-6 levels to inhibit inflammation | (46) |
| u-ADSCs | C57BL/6J mice |  | Atherogenic high fat (AT-HF) diet 4 weeks, 8 weeks |  | Splenic subcapsular  (splenic injection) | 1 × 106 cells or 7.5 × 105 cells | 2weeks、4weeks | Increased ratio of M1/M2 macrophages  Infiltration of CD11b+, F4/80+ and Gr-1+ inflammatory cells, improved fibrosis | (48) |
| ADSCs | Wistar rats |  | 1 g/mL LPS intervention |  | intraperitoneal injection | 1.5x106 cells | 6weeks | IL-1 and IL-6 inflammatory gene expression decreased  Transforming growth factor beta (TGF-β) decreased  ROS levels decreased | (50) |
| BMSCs | C57BL/6 mice |  |  |  | intravenous injection | 0.5 × 106 cells | 17weeks | TGF-β1 significantly reduces  inflammatory factors IL-1β, INF-γ, TNF-α and IL-6  and regulates lipid metabolism by upregulating the expression of ACO and CYP2E1 | (56) |
| BMSCs | Sprague Dawley rats | HepG2 cells |  |  | intravenous injections | 2 × 106  cells | 4weeks | Regulates Ca2+,  upregulates SERCA, downregulates BiP, ATF-4, p-eIF2a and CHOP expression, reduces endoplasmic reticulum stress  and inhibits pyroptosis (NLRP3, GSDMD/-N, caspase-1, p20, IL-1β and IL-18) | (58) |
| BMSC | C57BL/6 mice | HepG2 cells | Mitochondria expresses GFP |  | intravenous injections | 1 × 107 cells/kg | 24hor 48h  13 weeks | Decreased MDA, ROS and MMP levels  IP3R1 expression, regulating calcium homeostasis  Mitochondrial transfer increases OXPHOS activity and ATP levels, improves mitochondrial dysfunction, and maintains cellular bioenergetics | (59) |
| BMSC | Immunodeficient Pfp/Rag2-/- mice (C57BL6) |  |  |  | splenectomy | 0.9-1 × 106 cells | 1weeks | Upregulates PPARα and enhances the expression of genes involved in lipid utilization  Inhibits inflammation  Mitochondrial transfer provides oxidative capacity Reduces lipid load | (60) |
| Bone-derived | C57BL/6 mice |  |  |  | intravenous injection | 1 × 10 6  cells | 4weeks | Reduce inflammation and oxidative stress  Inhibit the proliferation of CD4+IFN-γ+ and CD4+IL-6+ lymphocytes and suppress the immune response | (62) |
| Bone-derived | C57BL/6 mice |  |  |  | intravenous injection | 1×106 cells | 7weeks | Reduces fat degeneration and lobular inflammation  Inhibits fibrosis  Reduces CD4+ T lymphocytes and inhibits immune response | (61) |
| MenSCs) | C57BL/6 mice | AML12cells, L02cells |  |  | intravenous injections | 5×105 cells | 11weeks | Secreted HGF inhibits Rnf186, inhibits insulin resistance and hepatic glycolipid metabolism  Targeting the AMPK-mTOR pathway  Increases p-Akt/p - Gsk3 β levels | (63) |
|  |  |  |  |  |  |  |  |  |  |
