# Supplementary material for: The therapeutic potential of different mesenchymal stem cells and their derived exosomes in metabolic dysfunction-associated steatotic liver disease
Source: Front Endocrinol (Lausanne). 2025 Apr 3;16:1558194. doi: 10.3389/fendo.2025.1558194 (PMC12003127; doi:10.3389/fendo.2025.1558194)
Supplement: Supplementary file 2 [file Table2.docx]

Supplementary Material

Supplementary Table 2.

| **Table 2. Effects of MSCs-Exo in MASH/MASLD** | | | | | | | | | | |
| --- | --- | --- | --- | --- | --- | --- | --- | --- | --- | --- |
| **Exo source** | **size** | **Animal model** | **In vitro model** | **Precondition** | **Route** | **dose/cell** | **follow-up period** | **Related exosome cargo** | **Effects** | **references** |
| HUC-MSCs) | 110 nm | C57BL/6 mice | L02cells,HFL1cells,AML12cells, HEK293T cells  、 | Knock down CAMKK1 | intravenous injections | 10 mg/kg  800 μg/ml | 4weeks,24h |  | Activates AMPK-mediated PPARα/CPT-1A and SREBP-1C/FASN signaling pathways to inhibit lipid deposition  MSC-ex enriched with CAMKK1  Reduces insulin resistance  Expression of inflammatory factors TNF-α, IL-1 and IL-6 | (69) |
| HUC-MSCs) | 72.22 nm | C57BL/6J mice | HepG2 cells  、 AML12cells |  | intravenous injections | 100μg/mouse  100μg/mL, 200μg/mL | 6 weeks,24h |  | Altered aberrant expression of lipid-related genes SREBP-1c, PPAR-α, Fabp5, CPT1α, ACOX and FAS  Reduced F4/80 + macrophages, CD11c + macrophages  TNF-α, IL-6, MDA, CYP2E1 and ROS  Increased Nrf2/NQO-1 pathway expression | (71) |
| UC-MSC | 59-73 nm |  | HepG2 cells, LX2 cells, HUVECs cells | mir17-5p Inhibitor |  | 100 μg/ml | 8days |  | Decrease in TGF-β 1 and inflammatory pathways (IL-1, IL-6, and TNF-α) to reduce liver inflammation  Reduces collagen and COL I, α-SMA deposition to promote liver repair | (73) |
| HUC-MSCs | 40 - 120 nm | C57BL/6 mice | hepatocytes |  | intravenous injections | 120 mg/mouse  2 mg/1 105cells | 24h  15 weeks | miR-24-3p | Attenuates lipid deposition, ROS production and inflammation, oxidative stress  Fatty acid synthesis molecules ACCa, FASN, SCD1 and PPARγ oxidative stress markers NOX2 and NOX4, as well as inflammatory signals TNF-a, IL-6, IL-1b and CCL2 decreased  Targeting the Keap-1/Nrf2 pathway | (72) |
| HUC-MSC | 96 nm | Sprague-Dawley rats | L-O2 cells | transfected with miR627-5p inhibitor | intravenous injections | 2 μg（cell）  100 μg（ animal ） | 24 h   2 months | miR-627-5p | Ameliorated liver injury, lipid deposition and glycolipid metabolism in vivo  Decreased the expression of G6Pc, PEPCK, FAS and SREBP-1c, and enhanced the expression of PPARα  Targeting FTO | (70) |
| Huc-MSCs | 110 nm | C57BL/6 mice | AML12cells |  | intravenous injections | 400 μg/ml（3ml）  2 mg/kg（ animal ） | 24-48h  4 weeks |  | Reduces lipid deposition, induces autophagy  Modulates AMPK/mTOR pathway and increases EI24 levels  Increased LC3BII/I ratio and significantly downregulated P62 protein levels | (53) |
| Huc-MSCs | 60 ~ 130 nm | Local strain of albino mice | HepG2 cells | Curcumin pretreatment | intravenous injections | 15μg / kg | 4 weeks /12 weeks |  | Improved lipid accumulation, MDA  Improvement of liver fibrosis, decrease of α-SMA, collagen-1 and MMP-1  Improve oxidative stress, inflammation, TNF-α, IL-6, IL-17  Decrease in ASK1 / JNK / BAX pathway gene expression, anti-apoptosis | (75) |
| ADSCs | 95.8 ± 1.2 nm | C57BL/6 mice | NCTC1469 cells  、 HEK293T cells  、 | Transfection of miR-223-3p mimic inhibitors | intravenous injections |  | 6 weeks | miR-223-3p | Reduces lipid accumulation, fibrosis  Targets E2F1 downregulation | (64) |
| BM-MSCs |  | Sprague-Dawley rats |  |  | intravenous injections | 15μg / kg 、 30μg / kg 、 120μg / kg | 6 weeks |  | Targeting miRNA-96-5p downregulates caspase-2 expression  and downregulates fatty acid synthesis (SREB1, 2, ACC) and lipid uptake (CD36); upregulates fatty acid oxidation (PPARα, CPT1)  Reduces the Bax/Bcl2 ratio and resists apoptosis  Mitophagy genes (Parkin, PINK1, ULK1, BNIP3L, ATG5, ATG7, ATG12) increased | (77) |
| BMSC-exos | 109.4 nm | C57BL/6 mice | 3T3-L1 cells |  | intraperitoneal injection | 50μg/mouse  10 and 20 μg·mL（cell） | 4 weeks  24h |  | Reduces metabolic disorders and inflammation  Reduces lipid accumulation and decreases leptin and FABP4  Activates the PI3K/AKT signaling pathway Regulates insulin sensitivity | (76) |
| pcMSCs | 100-150 nm | C57BL/6J mice |  | let-7i-5p miRNA inhibitor | intraperitoneal injection | 1 × 109 particles | 48h | let-7i-5p miRNA | Inflammation (TNF-α, IL-6, leptin) decreases, inhibits NF-κB and HIF-1α  Mitochondrial damage and dysfunction  Inhibits apoptosis | (78) |
|  |  |  |  |  |  |  |  |  |  |  |
